# *Opisthorchis viverrini*—Current Understanding of the Neglected Hepatobiliary Parasite

**DOI:** 10.3390/pathogens12060795

**Published:** 2023-06-02

**Authors:** Matthias Yi Quan Liau, En Qi Toh, Vishalkumar Girishchandra Shelat

**Affiliations:** 1 Lee Kong Chian School of Medicine, Nanyang Technological University, 11 Mandalay Road, Singapore 308232, Singapore; 2 Department of General Surgery, Tan Tock Seng Hospital, 11 Jalan Tan Tock Seng, Singapore 308433, Singapore; 3 Surgical Science Training Centre, Tan Tock Seng Hospital, 11 Jalan Tan Tock Seng, Singapore 308433, Singapore

**Keywords:** *Opisthorchis viverrini*, cholangitis, cholecystitis, cholelithiasis, advanced periductal fibrosis, cholangiocarcinoma, neglected tropical disease, surgical resection, liver transplantation, Lawa model

## Abstract

Opisthorchiasis due to *Opisthorchis viverrini* infection continues to be a significant public healthcare concern in various subregions of Southeast Asia, particularly in Thailand, Laos, Cambodia, Myanmar, and Vietnam. The main mode of transmission is via consumption of raw or undercooked fish, which is deeply embedded in the culture and tradition of the people living near the Mekong River. After ingestion, the flukes migrate to the bile ducts, potentially causing many hepatobiliary complications, including cholangitis, cholecystitis, cholelithiasis, advanced periductal fibrosis and cholangiocarcinoma. Several mechanisms of opisthorchiasis-associated cholangiocarcinogenesis have been proposed and elucidated in the past decade, providing insight and potential drug targets to prevent the development of the sinister complication. The gold standard for diagnosing opisthorchiasis is still via stool microscopy, but the advent of novel serological, antigen, and molecular tests shows promise as more convenient, alternative diagnostic methods. The mainstay of treatment of opisthorchiasis is praziquantel, while treatment of opisthorchiasis-associated cholangiocarcinoma depends on its anatomic subtype and resectability. Thus far, the most successful fluke control programme is the Lawa model based in Thailand, which raised awareness, incorporated education, and frequent surveillance of intermediate hosts to reduce transmission of opisthorchiasis. Development of vaccines using tetraspanins shows promise and is currently ongoing.

## 1. Introduction

*Opisthorchis viverrini*, commonly known as the Southeast Asian liver fluke, is a parasite that belongs to the family Opisthorchiidae [1]. It is endemic in Southeast Asia and is estimated to cause opisthorchiasis in more than 10 million people in just Thailand and Laos alone [2]. The impact of liver fluke infection on daily living can be significant. A study by Sayasone et al. found that individuals infected with a higher burden of adult worms report significant gastrointestinal symptoms such as right upper quadrant pain and abdominal discomfort [3]. In addition, chronic infection with *Opisthorchis viverrini* is a leading cause of bile duct cancer in Southeast Asia [4]. Despite the significant number of deaths (1200–1800/year) and disability-adjusted life years (DALYs) due to opisthorchiasis and *Opisthorchis viverrini*-associated cholangiocarcinoma (150–235 × 10^3^ DALYs) [5], opisthorchiasis remains a neglected tropical disease [6].

The development of cholangiocarcinoma associated with *Opisthorchis viverrini* infection also brings about a large socioeconomic burden on regions where the fluke is endemic. According to Muller et al., the financial impact of opisthorchiasis and cholangiocarcinoma in Thailand is estimated to be USD 120 million annually in healthcare costs and lost wages [7]. As opisthorchiasis is most prevalent in people of lower socioeconomic status, the financial impact on their families is even more significant. Despite the implementation of the Universal Health Coverage scheme to subsidize treatments for this group of patients, accessibility to care and affordability of treatment are unmet needs. Without attention and intervention, if status quo prevails, the socioeconomic gap in endemic regions is likely to widen and negatively impact these societies in future [8]. Therefore, prompt, effective, and efficient implementation of transmission controlling measures is necessary to reduce the global burden of *Opisthorchis viverrini*.

This is not to be confused with opisthorchiasis caused by *Opisthorchis felineus*, as well as hepatobiliary infections caused by other flukes such as *Clonorchis sinensis*, which also present similarly to opisthorchiasis [1]. In fact, *Clonorchis sinensis* was previously known as *Opisthorchis sinensis* from 1895 to 1907 [9]. Despite similarities in adult morphology and genetics, they vary greatly in geographical distribution, with *Opisthorchis felineus* endemic in Western Siberia [10], *Clonorchis sinensis* endemic in China, Korea, and Japan, whereas *Opisthorchis viverrini* is endemic in the Lower Mekong Subregion. *Clonorchis sinensis* and *Opisthorchis viverrini* are well known to increase the risk of cholangiocarcinoma in infected individuals and are classified as Group 1 carcinogens by the International Agency for Research on Cancer [11]. On the other hand, *Opisthorchis felineus* is not a recognized carcinogen (Group 3), though studies have shown its association with cholangiocarcinogenesis in hamsters and humans [12,13,14]. For example, a recent case-control study in Western Siberia by Fedorova et al. found that exposure to *Opisthorchis felineus* infection was strongly associated with cholangiocarcinoma development [14]. This review will focus on opisthorchiasis caused by *Opisthorchis viverrini*, and serve as an update to previous reviews including those by Sripa et al. in 2011 [15] and Gundamaraju et al. in 2014 [16]. It will also highlight the hepatobiliary manifestations, advancements in detection and treatment of *Opisthorchis viverrini*-associated cholangiocarcinoma in the past decade.

## 2. Methods

PubMed and Web of Science were searched from inception to April 2023 using the following keywords: “*Opisthorchis viverrini*”, “epidemiology”, “morphology”, “life cycle”, “hepatobiliary”, “pathogenesis”, “genomics”, “transcriptome”, “proteomics”, “cholangiocarcinoma”, “serology”, “antigen test”, “polymerase chain reaction”, “imaging”, “treatment”, “liver transplant”, “chemotherapy”, “Lawa model”, and “vaccine”. In similar studies or studies with progressive development, only the most updated information was extracted. Information retrieved from relevant articles on the epidemiology, morphology, life cycle, hepatobiliary manifestations, pathogenesis, omics, diagnostic and treatment modalities, and prevention strategies for *Opisthorchis viverrini* infection are presented below.

## 3. Epidemiology

*Opisthorchis viverrini* is endemic in the Lower Mekong Subregion, particularly in Thailand, Laos, Cambodia, Myanmar, Vietnam and China [17,18], where the consumption of raw or undercooked cyprinid fish is a long-standing and common dietary practice among people who live near wetlands and water bodies [19,20,21]. In Thailand, it is estimated that over 6 million people are infected with the liver fluke *Opisthorchis viverrini*, making it a major public health concern in the country [22]. The prevalence of infection varies between regions, with historical *Opisthorchis viverrini* infection rates reported to vary between 4.6% and 60% in 2009 [23]. Despite efforts undertaken by Thailand’s Ministry of Public Health to eradicate *Opisthorchis viverrini* infection, the highest rates are still observed in the north-eastern provinces such as Nakhon Phanom, with a prevalence of 24% in 2019 [24]. Opisthorchiasis is also a concern in neighbouring countries, where prevalence rates of 54.8% and 7.7% have been reported in Laos in 2015 [25] and in Cambodia in 2012, respectively [26]. Globalisation also threatens to spread the disease further across Southeast Asia [2]. On an interesting note, Mungmunpuntipantip et al. described a lower incidence of coronavirus disease 2019 (COVID-19) in areas with higher prevalence of opisthorchiasis in Thailand, though no clinical evidence of the underlying pathophysiology has been presented [27].

## 4. Morphology

*Opisthorchis viverrini* is a small, flatworm parasite that ranges in size from 5 to 10 mm in length and 1 to 2 mm in width [28]. The adult worm is transparent and has a thin, lancet shape. It also has a ventral and an oral sucker, which helps it to adhere to the lining of the biliary ducts. The fluke is monoecious as it possesses lobed testes and an ovary, which produces eggs that are excreted in the faeces of the host [29].

The eggs of *Opisthorchis viverrini* are oval-shaped and measure about 30 µm in length and 12 µm in width. On the other hand, the metacercariae are spherical, and they have a clear and thin wall. The size of the cysts that contain them is about 130 × 170 µm. Inside the cysts, the juveniles have dark granules in the excretory bladder and scattered brown pigments in the body. The oral and ventral suckers are of equal size [29].

## 5. Life Cycle

**Figure 1:**
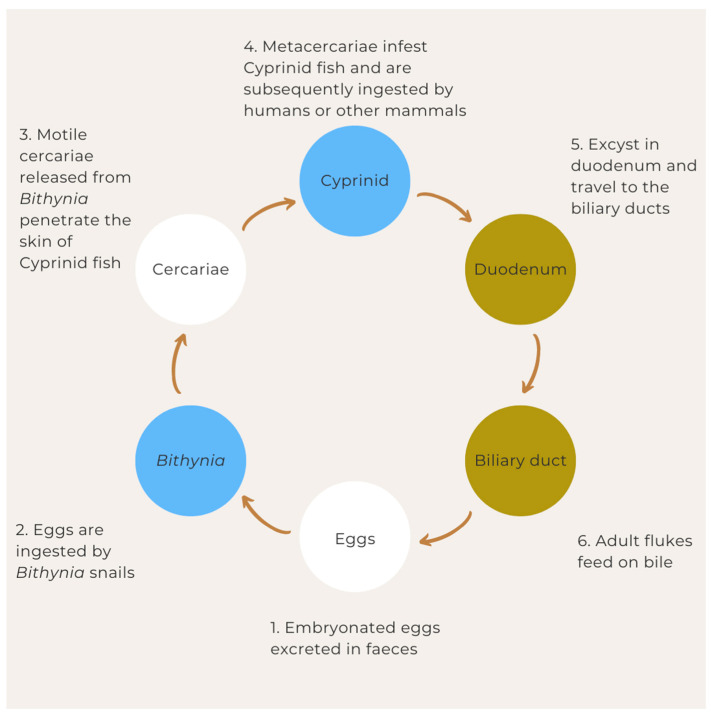
Life cycle of *Opisthorchis viverrini*.

The life cycle of *Opisthorchis viverrini* is complex and involves two intermediate hosts (snail and freshwater fish) and a definitive host (mammal) (Figure 1). It begins with the production of eggs by adult worms residing in the biliary ducts of the definitive host, which is typically a human or other mammals, such as cats and dogs. The embryonated eggs are excreted in the faeces of the host and are released into the environment [28].

The eggs of *Opisthorchis viverrini* are ingested by freshwater snails of the genus *Bithynia*, which are the first intermediate hosts [30]. They then hatch into miracidia, which develop into sporocysts. The sporocysts give rise to rediae, which subsequently produce cercariae [28]. The cercariae are released from the snail into the water. They are motile and infect the second intermediate host, which is usually a freshwater fish of the Cyprinidae family, such as carp [31]. The cercariae penetrate the skin of the fish and migrate to the muscle tissue, where they encyst and develop into metacercariae. When humans or other mammals consume raw or undercooked fish infected with metacercariae, the juvenile worms are released from the cysts and migrate to the bile ducts, where they mature into adult flukes, completing the life cycle. The adult worms feed on the host’s biliary cells [32] and can live for more than 10 years [30].

## 6. Omics

In 2014, Young et al. characterized the draft genome and transcriptomes of *Opisthorchis viverrini* [6]. In addition, many studies on the proteomics of the fluke have been published in the past decade as well [33]. Together, they provide insight into how the fluke survives in the bile ducts and its interactions with the host.

The adult fluke is exposed to a low-oxygen environment in the bile ducts. To adapt to this, the fluke increases transcription of genes responsible for haemoglobin production [6]. In addition, *Opisthorchis viverrini* protects itself from toxins, drugs, and carcinogens by expressing antioxidants such as glutathione-S-transferases [6,34]. Further, *Opisthorchis viverrini* also produces cathepsin F peptidases which degrade immunoglobulins, enabling it to evade the host immune system [6].

More importantly, omics data are also relevant to the understanding of *Opisthorchis viverrini*-associated cholangiocarcinogenesis, and has applications in predicting diagnostic markers, treatment, and vaccines, which are described in subsequent sections.

## 7. Hepatobiliary Opisthorchiasis

The hepatobiliary manifestations of opisthorchiasis are primarily due to the inflammation and damage to the bile ducts. The initial phase of the infection is asymptomatic, but chronic infection can lead to various hepatobiliary complications, including cholangitis, cholecystitis, cholelithiasis, advanced periductal fibrosis, and cholangiocarcinoma [35,36,37].

The initial migration of *Opisthorchis viverrini* to the extrahepatic and intrahepatic bile ducts and feeding by suckers induce physical damage to the ductal epithelium [38]. Activation of host inflammation also occurs via interleukin-6 production [39]. These mechanisms result in the proliferation and desquamation of ductal epithelium in the early stages of the disease. This is followed by periductal fibrosis, leading to biliary obstruction and the progressive dilatation of the bile ducts [36]. The irritation and inflammatory mediators produced cause inflammation of the bile ducts, resulting in cholangitis when severe. The biliary obstruction increases intrabiliary pressure, which increases the permeability of the bile ductules. This allows translocation of toxins and bacteria from the biliary tract into the portal circulation. In severe cases, chronic *Opisthorchis viverrini* infection may result in recurrent pyogenic cholangitis [40]. Acute suppurative cholangitis may also arise when the extrahepatic bile ducts are occluded by masses of dead organisms and mucin, in turn precipitating ascending cholangitis [16].

Adult *Opisthorchis viverrini* flukes also shed an array of excretory–secretory (ES) products and crude somatic (CS) antigens into the biliary epithelium of the biliary ducts and gallbladder during infection [41]. These have the ability to activate mucosal inflammatory cascades, resulting in cholecystitis [42]. For example, a study by Jittimanee et al. showed that CS and ES antigens induce mRNA expressions and an increase in protein levels of CD80 and MHC class II, both of which play an important role in dendritic cell maturation and T cell activation, leading to a pro-inflammatory state. However, the same antigens are also found to be able to stimulate the production of large amounts of interleukin-10 (IL-10) and transforming growth factor beta (TGF-β) concurrently, which are immunosuppressive in nature [43].

**Figure 2:**
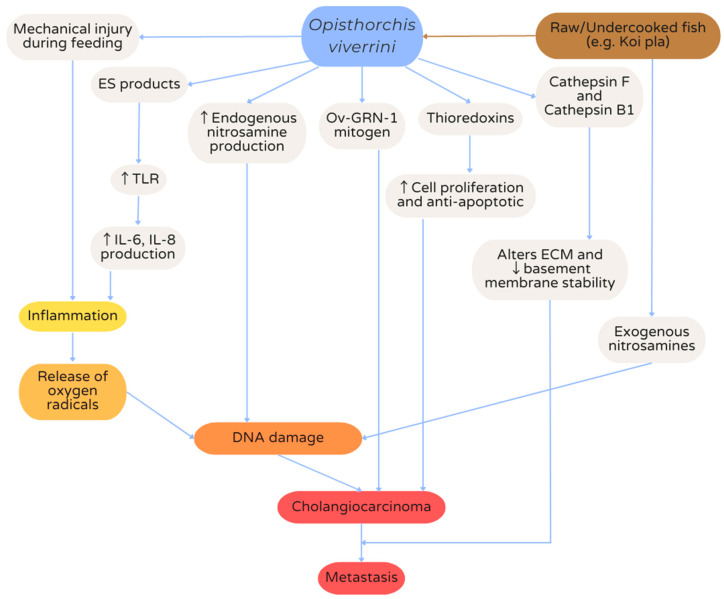
Mechanisms of cholangiocarcinogenesis from *Opisthorchis viverrini* infection. ES, excretory–secretory; *Ov*-GRN-1, *Opisthorchis viverrini* granulin-like substance; TLR, Toll-like receptor; IL, interleukin; ECM, extracellular matrix; DNA, deoxyribonucleic acid.

There are many factors that contribute to *Opisthorchis viverrini*-associated cholangiocarcinogenesis (Figure 2). Firstly, the action of suckers during feeding and the movement of the flukes cause mechanical injury to the ductal epithelial cells. In addition, *Opisthorchis viverrini* produces proteases such as cathepsin F and cathepsin B1, which contribute to tissue destruction due to their extracellular protein degradation activity [33]. Excretory–secretory products from *Opisthorchis viverrini* also result in a proinflammatory response, leading to upregulation of Toll-like receptors (TLRs) and increased production of interleukins 6 and 8, which in turn activate downstream chemokines which contribute to cholangiocarcinogenesis [41]. Chronic injury to ductal tissues from these mechanisms leads to oxidative DNA damage and mutation in the long run, increasing the risk of cholangiocarcinoma development. *Opisthorchis viverrini* also releases mitogenic and anti-apoptotic factors such as granulin-like growth factor *Ov*-GRN-1 and thioredoxins, which stimulate cell proliferation and lead to the development of cholangiocarcinoma [32,44]. Further, exogenous nitrosamines are found in the food typically contaminated with *Opisthorchis viverrini* metacercariae [45]. *Opisthorchis viverrini* infection itself also drives an increase in endogenous nitrosamine production, which is known to be carcinogenic and contributes to cholangiocarcinogenesis [46]. Finally, the proteases cathepsin F and cathepsin B1 may also alter the local extracellular matrix environment through tissue destruction, resulting in basement membrane instability and encouraging invasion and metastasis of the cholangiocarcinoma [47]. As a whole, opisthorchiasis-associated cholangiocarcinoma is extremely invasive, develops rapidly, has high metastatic potential and very poor prognosis [15].

## 8. Diagnosis

In individuals who have consumed raw or uncooked fish from endemic areas and present with clinical symptoms such as right upper quadrant pain, nausea, vomiting, anorexia, or jaundice, the diagnosis of opisthorchiasis should be suspected. The identification of eggs in stool is considered the gold standard and the primary means of diagnosing opisthorchiasis, with the eggs typically appearing in stool 3–4 weeks post-exposure. However, the eggs are not always detectable in the faeces, particularly in individuals with mild infections or biliary obstruction [48].

Alternative diagnostic tests such as serologic, antigen and molecular tests are in development but are not yet widely available, and these are summarized in Table 1.

### 8.1. Serological Diagnosis

Serological tests for opisthorchiasis are primarily based on enzyme-linked immunosorbent assays (ELISA). An immunochromatographic test kit based on the principles of ELISA has been developed using soluble excretory–secretory antigen from adult *Opisthorchis viverrini* flukes by Sadaow et al. When tested with 236 serum samples of healthy volunteers, patients with opisthorchiasis and patients with other helminth infections, the developed test kit demonstrated high sensitivity, specificity, positive, and negative predictive values of 100%, 98.3%, 97.9%, and 100%, respectively [50]. Phupiewkham et al. also developed immunochromatographic test kits using somatic antigens with IgG and IgG4 conjugates which showed lower sensitivity but higher specificity [49]. Despite advancements in serological detection methods, cross-reactivity with several other parasitic infections still remains a significant limitation to implementation. These methods also have limited utility as they do not aid in differentiating between past and present infections, due to the long half-life of the antibodies formed in response [15].

### 8.2. Antigenic Diagnosis

Urine antigen detection is another promising approach for detecting opisthorchiasis. A study conducted in Thailand involving over 1000 individuals found that the urine antigen detection using monoclonal antibody-based enzyme-linked immunosorbent assay (ELISA) has high diagnostic accuracy and is more sensitive than stool microscopy [55]. Antigen tests may become negative 4 weeks after treatment, suggesting that they may aid in distinguishing current from previous infections [56].

To improve the sensitivity and specificity of urine antigen tests from traditional ELISA assays, gold nanoparticles were used in the development of an ELISA assay in a study by Taron et al. The novel assay showed a significant improvement in sensitivity and specificity of 93.81% and 91.34%, respectively, and was successfully used to detect *Opisthorchis viverrini* antigens in urine samples [52]. Recently, a portable point-of-care device for diagnosis of *Opisthorchis viverrini* antigen in urine samples has also been developed. This device consists of a portable fluorometer based on smartphone devices, as well as a fluorescent assay enhanced with aqueous micelles of non-ionic surfactant. Further testing of the proposed device in an endemic area showed high sensitivity, specificity and accuracy of 84.88%, 89.66%, and 86.14%, respectively, suggesting its viability in point-of-care diagnostics [51].

### 8.3. Molecular Diagnosis

Molecular methods such as polymerase chain reaction (PCR) have also been developed by Pumpa et al. that are effective in detecting mild infections [54]. This involves detecting *Opisthorchis viverrini* eggs by targeting three genes, internal transcribed spacer-2 (ITS-2), cytochrome oxidase subunit 1 (*cox1*), and cytochrome b (*cyb*). Another study by Phadungsil et al. also utilized PCR and targeted *Opisthorchis viverrini* NADH dehydrogenase subunits to detect eggs, with high sensitivities and specificities of up to 100% across 166 stool samples [53]. Overall, PCR has shown some potential in playing a role in the assessment of cure, re-infection and characterising the endemic range of these flukes in East Asia [15], but the need of special equipment often limits their widespread implementation.

### 8.4. Imaging Modalities

Ultrasound can be used to aid visualisation of the disease. Firstly, several hepatobiliary abnormalities related to opisthorchiasis have been detected with ultrasound. The height of the left hepatic lobe adjusted for body weight in females and those below 45 years old increased with the burden of infection, while it was reduced in heavily infected males and those of an older age group. Increased echoes and periportal echoes have also been observed on the hepatic parenchyma. Further, sludge and irregular gallbladder walls are also noted in a significant proportion of cases [57].

Secondly, ultrasonography can be used in following up on the patients for any signs of complications. In particular, ultrasound can detect advanced periductal fibrosis (APF). In chronic opisthorchiasis, peripheral intrahepatic bile ducts become dilated and thickened from fibrotic deposition, which appears on ultrasound as echogenic nodules with an anechoic centre. Persistent APF has been proposed to be a risk factor for progression to cholangiocarcinoma [15].

Computed tomography (CT) may also aid visualisation of the disease, as it shows good detail of liver parenchyma, associated ducts, and any dilatation of the biliary tree. The degree of dilatation should suggest the degree and duration of obstructive jaundice. If the disease is complicated by cholangiocarcinoma, small nodular tumour masses may also be visible in the dilated bile duct. As they progress, low attenuation areas can be seen around porta hepatis or in the liver parenchyma and may cause more obstruction. Lymphadenopathy in porta hepatis is also commonly observed [58].

Magnetic resonance imaging (MRI) can also be used in conjunction with ultrasonography and/or computed tomography to evaluate the progression of the disease. The degree of dilatation of intra- and extrahepatic ducts corresponds to the amount of inflammation and fibrosis, which are visualized as hyperintense signals on T2-weighted MRI [59]. Apart from that, MRI is the most sensitive and specific imaging modality for the diagnosis of premalignant cholangiocarcinoma lesions including biliary intraepithelial neoplasia and intraductal papillary neoplasm of the biliary tract. These appear as focal bile duct dilatation and focal non-bile-duct excretion of biliary contrast agents [60]. In addition, MRI is useful in pre-operative planning as it allows for the assessment of vascular involvement and hence resectability, as well as pre-operative vascular road mapping for planning extended liver resections [61].

Finally, endoscopic retrograde cholangiopancreatography (ERCP) may be used as a theranostic tool to visualize the bile ducts and relieve biliary obstruction. During fluoroscopy, adult *Opisthorchis viverrini* flukes may present as small, elongated filling defects in the biliary ducts up to 10 mm in length. Dilatation of the small and medium-sized intrahepatic ducts are more common; however, extrahepatic ducts may also be dilated in severe infection with a heavy burden of adult flukes [62].

## 9. Treatment

### 9.1. Eradication of Parasites

The treatment of hepatobiliary opisthorchiasis involves the use of anthelmintic drugs, such as praziquantel and tribendimidine. The first-line treatment for *Opisthorchis viverrini* infection is praziquantel with a dosage of 25 mg/kg three times a day for 2 to 3 consecutive days as recommended by the World Health Organization. Alternatively, a single dose of 40 mg/kg praziquantel can be used. All cases of confirmed *Opisthorchis viverrini* infection and suspected cases in endemic regions should be treated regardless of whether they are symptomatic to reduce potentially severe complications such as recurrent pyogenic cholangitis and cholangiocarcinogenesis [63].

Alternatively, tribendimidine is suggested to be at least as efficacious as praziquantel in the treatment of *Opisthorchis viverrini* infections [64,65,66]. In a randomized controlled trial involving more than 600 patients, tribendimidine was shown to have a cure rate slightly lower than praziquantel of 94% compared to 97%, but it has a similar egg reduction rate of 99.9% to praziquantel. Tribendimidine is also associated with fewer adverse events and may serve as a valuable alternative to praziquantel [65].

### 9.2. Treatment of Co-Infection (Helicobacter pylori)

*Opisthorchis viverrini* infections lead to hepatobiliary manifestations, including advanced periductal fibrosis (APF), which is correlated with the risk of cholangiocarcinoma. The existing literature proposes that a carcinogenic bacterium, *Helicobacter pylori* (*H. pylori*), also contributes to the development of cholangiocarcinoma by enhancing the severity of hepatobiliary abnormalities [67]. A study by Hang et al. found that even after completion of therapy with praziquantel, patients with co-infection by *H. pylori*, especially cagA-positive strain, continued to have persistent APF [68]. Hence, this suggests the need for concurrent *H. pylori* treatment for better outcomes.

### 9.3. Treatment of Symptoms

Patients infected by *Opisthorchis viverrini* often present with hepatobiliary symptoms and should be treated. Cholecystectomy is indicated in patients with isolated cholecystitis; early laparoscopic cholecystectomy is associated with better patient outcomes [69,70] and is considered gold standard treatment. Further, decompression of the biliary tract and drainage of the abdominal cavity via percutaneous transhepatic biliary drainage (PTBD) is required in patients presenting with cholangitis to prevent biliary peritonitis in the postoperative period [71]. ERCP is also another useful modality to treat choledocholithiasis associated with *Opisthorchis viverrini* infection via endoscopic sphincterotomy [72]. Although endoscopic extraction of worms has led to rapid resolution of symptoms in other parasitic infections such as biliary ascariasis, endoscopic extraction of adult *Opisthorchis viverrini* flukes has been not reported to date [73].

### 9.4. Treatment of Opisthorchis viverrini-Associated Cholangiocarcinoma

The treatment of *Opisthorchis viverrini*-associated cholangiocarcinoma depends on the anatomic subtype, which includes intrahepatic, perihilar and distal extrahepatic cholangiocarcinoma [74]. Early-stage tumours are amenable to surgical resection or liver transplantation, whereas palliative chemotherapy is the mainstay of treatment for advanced-stage disease. Several targeted and immune-directed therapies are currently in development and have shown promising results in phase II trials [75].

#### 9.4.1. Surgical Resection

Prior to liver resection, staging laparoscopy is recommended to evaluate for any occult peritoneal metastases. If present, these patients should be spared an unnecessary laparotomy due to unresectable disease [76]. Liver resection for intrahepatic cholangiocarcinoma is potentially curative if margin negative (R0) resection can be achieved [75]. On the other hand, surgical resection with a positive resection margin (R1) is associated with a decrease in the 5-year survival from 32.2% to 13.1% and a drop in median recurrence-free survival from 12.4 months to 7.4 months [77].

In perihilar cholangiocarcinoma, liver resection with negative margin is associated with a 5-year survival of 67.1% [78]. In addition, patients with locally advanced Bismuth type IV perihilar cholangiocarcinoma, which has traditionally been categorized as unresectable, have improved 5-year survival of 32.8% from 1.5% post-resection [79]. A new modification of the 8th American Joint Committee on Cancer (AJCC) staging system, the Khon Kaen University (KKU) staging system for perihilar cholangiocarcinoma, has been proposed recently. It uses growth patterns, histological grading, and lymph node and distant metastases to prognosticate the overall survival, and a prospective study is underway to evaluate its effectiveness as a prognostic tool [80].

Pancreaticoduodenectomy is the treatment of choice for surgically resectable distal cholangiocarcinoma. Prognostic factors post-pancreaticoduodenectomy of distal cholangiocarcinoma include size of tumour, lymph node status, growth patterns and resection margin status [81,82]. Achieving a negative margin resection has been found to significantly increase overall survival from 9 months to 48 months [83].

#### 9.4.2. Liver Transplantation

Liver transplantation for intrahepatic cholangiocarcinoma has been a subject of controversy due to the high incidence of recurrence after transplantation and the scarcity of donor organs. However, recent studies have found that liver transplantation can yield better outcomes in a selected group of patients with smaller tumours and favourable tumour biology [84]. For example, a study by McMillan et al. saw that patients who underwent liver transplantation had an overall survival of 100%, 71% and 57% at 1, 3 and 5 years [85].

In perihilar cholangiocarcinoma, neoadjuvant chemotherapy followed by liver transplantation has been found to provide a survival benefit with overall survival at 2-years at 65–70% and 5-year recurrence-free survival at 47–68% [86]. Elevated carbohydrate antigen 19–9 (CA 19–9) and portal vein encasement are identified as predictors of recurrence post-transplant [87].

#### 9.4.3. Locoregional Therapy

Locoregional therapies such as thermal ablation, transarterial chemoembolization (TACE), selective internal radiation therapy (SIRT), chemotherapy hepatic arterial infusion (HAI), and external beam radiotherapy (EBRT) can be used to treat unresectable liver-only intrahepatic cholangiocarcinoma [88]. A pooled analysis conducted by Edeline et al. found the overall survival of patients who underwent locoregional therapies for intrahepatic cholangiocarcinomas to be 30.2 months for thermal ablation, 15.9 months for TACE, 14.1 months for SIRT, 21.3 months for HAI and 18.9 months for EBRT. Overall survival was also found to be higher in patients who were treated with systemic chemotherapy before TACE, SIRT, or HAI [89].

#### 9.4.4. Systemic Therapy

Systemic therapies used to treat cholangiocarcinoma include adjuvant therapy post-surgical resection and palliative chemotherapy. A randomised controlled multicentre phase III trial (BILCAP study) showed that the use of capecitabine as adjuvant therapy had an overall improved survival of 53 months as compared to 36 months in the observation group [90]. Based on these findings, the American Society of Clinical Oncology (ASCO) clinical practice guideline recommends the use of adjuvant capecitabine chemotherapy for a duration of 6 months post-resection. In addition, patients with positive resection margins may be offered chemoradiotherapy [91]. In patients with advanced-stage disease, palliative chemotherapy options are available. The Advanced Biliary Cancer-02 (ABC-02) phase III trial involving 410 patients showed that a cisplatin plus gemcitabine regime conferred a longer survival period of 11.7 months as compared to 8.1 months when gemcitabine was used alone, without the addition of substantial toxicity [92]. The ABC-06 phase III clinical trial conducted showed that the use of folinic acid, fluorouracil, and oxaliplatin chemotherapy regime as second-line treatment in patients who became unresponsive to the first-line cisplatin-plus-gemcitabine regime resulted in an improvement in survival from 5.3 months to 6.2 months [93].

Numerous studies on targeted therapy for *Opisthorchis viverrini*-associated cholangiocarcinoma are in progress. Loilome et al. identified PRKARIA (a regulatory substrate of protein kinase A) and MARCKS (a substrate of protein kinase C) as genes which are involved in cholangiocarcinogenesis and metastases [94,95,96]. These findings subsequently led to the investigation of the roles of other protein kinases in cholangiocarcinogenesis. One such study noted the overexpression of epidermal growth factor receptor (EGFR) in patients with *Opisthorchis viverrini*-associated cholangiocarcinoma and also found that cholangiocarcinoma cells from these tissues showed inhibited cell growth and metastatic potential after treatment with nimotuzumab [97]. Another study investigated the use of a highly selective pan-class I phosphatidylinositol 3-kinase (PI3K) inhibitor, buparlisib, to target the PI3K/RAC serine/threonine-protein kinase (Akt) pathway, which has been implicated in the development of *Opisthorchis viverrini*-associated cholangiocarcinoma [98]. The in vivo study on mice models led to a reduction in tumour size without substantial signs of toxicity, suggesting that buparlisib is a possible therapeutic agent [99]. More work needs to be carried out to develop specific inhibitors for targeted treatment of *Opisthorchis viverrini*-associated cholangiocarcinoma [100].

## 10. Prevention

Preventing opisthorchiasis requires a multifaceted approach that involves educating the public about the risks of consuming raw or undercooked fish, implementing screening programmes and mass treatment in regions where opisthorchiasis is prevalent. Preventing the acquisition of the disease and subsequent transmission is a cornerstone of the management of opisthorchiasis. Although the first-line treatment for opisthorchiasis is oral administration of praziquantel, it will not reliably reverse periductal fibrosis and inflammation. This implies that it may not be effective in preventing the dreaded sequelae of cholangiocarcinoma [15].

In a more recent development, vaccines against *Opisthorchis viverrini* have been tested in hamster models, but none are available for human use currently [75].

### 10.1. Lawa Model

The most successful and recognised liver fluke control programme implemented in Thailand is the Lawa model, which integrates the EcoHealth and One Health approaches to tackle *Opisthorchis viverrini* transmission. First, a treatment programme with intensive education was created targeting residents of different age groups to raise awareness of the causes of the *Opisthorchis viverrini* infection. In addition, knowledge on liver fluke infections and liver cancer were integrated into schools’ science curriculum, to empower students to remind family members not to consume raw or undercooked fish. Second, community hospital staff and volunteers were trained to oversee the implementation of the liver fluke control program in their village. Third, frequent surveillance of *Opisthorchis viverrini* infections and infection rates of intermediate hosts (Cyprinid fish and *Bithynia* snails) were conducted to study and investigate environmental conditions which promote fluke transmission [101,102,103].

A decade after the implementation of the Lawa model, the prevalence of *Opisthorchis viverrini* infection fell from a baseline of 60% to below 10% in all villages along the Lawa lake. After 3 years of implementation, *Opisthorchis viverrini* was also not detected in any school children. In addition, the residents also showed improved knowledge, attitudes, and practices with regards to fluke control measures. The prevalence of Cyprinid fish infected with metacercariae in the lake also decreased from 70% to less than 1%. Furthermore, no *Bithynia* snails were found to be infected with *Opisthorchis viverrini* as compared to the previous 0.18% [102]. Components of the successful Lawa model could thus be further adapted in other endemic regions to bring down *Opisthorchis viverrini* infections in the future.

### 10.2. Vaccines

Although there is currently no vaccine for opisthorchiasis infection in humans, a few subunit vaccines have been trialled in hamster models. Current efforts in vaccine development are primarily focused on targeting the uptake of extracellular vesicles (EVs) produced by *Opisthorchis viverrini* through the use of antibodies and disrupting parasite–host interactions. Tetraspanins, which are found in the tegmental membranes of *Opisthorchis viverrini*, are also found in EV membranes and they play a role in vesicle formation and uptake by host cells. In hamster models, vaccination with a recombinant EV tetraspanin (rO*v*-TSP-2) induced strong antibody responses crucial for protection against *Opisthorchis viverrini*, leading to a decrease in both worm and egg burden. In addition, the generated antibodies also reduced the uptake of fluke EVs by cholangiocytes, interrupting host–parasite interactions which could reduce the survivability of the fluke [104,105]. Another study developed a recombinant chimeric form of the large extracellular loop of O*v*-TSP-2 and *Opisthorchis viverrini* leucine aminopeptidase, and its use in vaccinated hamster models significantly reduced worm burden by 27% [106]. These promising studies suggest that recombinant tetraspanins, possibly fused with proteins involved in host–parasite interactions, could be used to develop effective vaccines against *Opisthorchis viverrini* in the future.

## 11. Conclusions

In conclusion, *Opisthorchis viverrini*, despite its small size, has the potential to cause grave damage to the hepatobiliary system, ranging from serious infection to malignancy. This not only brings severe consequences to the people’s health, but also has socioeconomic implications for them, all of which, however, could be prevented with the appropriate measures in place. These include prevention, early diagnosis, adequate treatment, and close monitoring for the development of serious sequelae of the infection, if any. Therefore, although there has already been some improvement over the years, more work is still required to boost ongoing efforts, given the high prevalence and gravity of the disease.

## Figures and Tables

**Table 1:** Serological, antigenic, and molecular diagnostic tests for *Opisthorchis viverrini*. IgG, immunoglobulin G; NADH, nicotinamide adenine dinucleotide (reduced form).

Study	Target	Results
**Serological Diagnostic Tests**		
Phupiewkham 2021 [49]	Somatic antigens of adult *Opisthorchis viverrini* with IgG and IgG4 conjugates	IgG Sample size: 332 Sensitivity: 86.6% Specificity: 89.5% Positive predictive value: 82.9% Negative predictive value: 91.9% IgG4 Sample size: 332 Sensitivity: 75% Specificity: 98.4% Positive predictive value: 96.6% Negative predictive value: 87%
Sadaow 2019 [50]	Excretory–secretory antigen from adult *Opisthorchis viverrini*	Sample size: 236 Sensitivity: 100% Specificity: 98.3% Positive predictive value: 97.9% Negative predictive value: 100%
**Antigenic Diagnostic Tests**		
Taron 2021 [51]	Portable smartphone-based fluorescent enzyme-linked immunosorbent assay	Sample size: 440 Sensitivity: 84.88% Specificity: 89.66% Positive predictive value: 95.82% Negative predictive value: 67.97%
Taron 2020 [52]	Enzyme-linked immunosorbent assay enhanced with gold nanoparticles	Sample size: 390 Sensitivity: 93.81% Specificity: 91.34% Positive predictive value: 81.54% Negative predictive value: 97.31%
**Molecular Diagnostic Tests**		
Phadungsil 2021 [53]	*Opisthorchis viverrini* NADH dehydrogenase subunits (*OvNad*1, *OvNad*2, *OvNad*4 and *OvNad*5)	Sample size: 75 *OvNad*1 sensitivity: 64.00% *OvNad2* sensitivity: 88.00% *OvNad4* sensitivity: 80.00% *OvNad*5 sensitivity: 100.00%
Pumpa 2021 [54]	Internal transcribed spacer-2 (ITS-2), cytochrome oxidase subunit 1 (*cox*1), and cytochrome b (*cyb*)	Sample size: 26 ITS-2 sensitivity: 76.9% *cox*1 sensitivity: 96.2% *cyb* sensitivity: 100%

## Data Availability

Not applicable.
